# Effect of a single dry needling session in temporomandibular disorders

**DOI:** 10.1080/07853890.2021.1896460

**Published:** 2021-09-28

**Authors:** Paula Moleirinho-Alves, Pedro Cebola, André Mariz, Almeida

**Affiliations:** a Escola Superior de Saúde Egas Moniz, Almada, Portugal; b Instituto Universitário Egas Moniz, Almada, Portugal; c Centro de Investigação Interdisciplinar Egas Moniz, Almada, Portugal; d Pratica Clínica Privada

## Abstract

**Introduction:**

Temporomandibular disorders embrace a heterogeneous group of pathologies with manifestations in the orofacial region, head and neck. They are defined as a group of musculoskeletal and neuromuscular conditions that involve the temporomandibular joint, the masticatory muscles and the associated structures through interactions and reciprocal influences. They may present one or more signs or symptoms: orofacial pain, pain in the masticatory muscles or a combination of both [1]. According to available literature, pain tolerance threshold at pressure and pain intensity decrease after dry needling intervention [2]. The aim of the present study is to evaluate the effect of a single dry needling session in patients with temporomandibular muscle disorder with pain.

**Material and methods:**

a longitudinal quasi-experimental study with 26 patients with a diagnosis of temporomandibular muscle dysfunction (group I), on the masseter and temporal muscles, according to the Research Diagnostic Criteria for temporomandibular disorder. Patients were randomly picked up from the waiting list of the physiotherapy appointment at Clinica Egas Moniz (CEA) and randomised into two groups: 13 in the experimental group (G1) and 13 in the control group (G2). The groups were evaluated simultaneously but were not submitted to any intervention. The pain tolerance threshold was evaluated at pressure (2 kg), pain intensity measured with numeric pain rating scale (NPRS), before (T0) and after (T1) the intervention, and one week after (T2) the intervention. The intervention consisted of applying the dry needling technique to the painful points of the masseter. Statistical analysis performed in SPSS with a significance level of 5%. All the assumptions of the Helsinki Declaration have been fulfilled and a informed consent for clinical case of Clinica Dentária Egas Moniz approved by the ethic commission of Instituto Universitário Egas Moniz.

**Results:**

G1 group was formed by 12 female and 1 male subjects with an average and standard deviation of 22.78 ± 3.75. G2 group was formed by 13 female with an average and standard deviation of 23.42 ± 2.45. Pain tolerance threshold at pressure values increased from T0 to T2 in G1 group (*p* < .001) with averages and standard deviations respectively for: T0 8.34 ± 1.75; T1 79,886 ± 2.10; T2 1.78 ± 0.79. The pain intensity values decreased in T2 when compared to T0 in the G1 group (*p* < .001) with averages and standard deviations respectively for: T0 7.56 ± 1.36; T1 7.86 ± 1.52; T2 0.89 ± 0.72.

**Discussion and conclusion:**

Dry needling promotes an increase in pain tolerance threshold values and a decrease in pain intensity values in patients with temporomandibular muscle disorder after a single session, reinforcing their importance in the treatment of themselves.
